# Endothelial deletion of PKCδ prevents VEGF inhibition and restores blood flow reperfusion in diabetic ischemic limb

**DOI:** 10.1177/1479164121999033

**Published:** 2021-03-15

**Authors:** Laura Croteau, Clément Mercier, Étienne Fafard-Couture, Alexandre Nadeau, Stéphanie Robillard, Valérie Breton, Andréanne Guay, Farah Lizotte, Marc-Antoine Despatis, Pedro Geraldes

**Affiliations:** 1 Research Center of the Centre Hospitalier Universitaire de Sherbrooke, Sherbrooke, QC, Canada; 2 Department of Surgery of the Centre Hospitalier Universitaire de Sherbrooke, Sherbrooke, QC, Canada; 3 Department of Medicine, Division of Endocrinology, Université de Sherbrooke, Sherbrooke, QC, Canada

**Keywords:** Diabetes, peripheral arterial disease, PKCδ, vascular endothelial growth factor, SH2 domain-containing phosphatase 1

## Abstract

**Aims:**

Peripheral artery disease is a complication of diabetes leading to critical hindlimb ischemia. Diabetes-induced inhibition of VEGF actions is associated with the activation of protein kinase Cδ (PKCδ). We aim to specifically investigate the role of PKCδ in endothelial cell (EC) function and VEGF signaling.

**Methods:**

Nondiabetic and diabetic mice, with (*ec-Prkcd*^−/−^) or without (*ec-Prkcd*^f/f^) endothelial deletion of PKCδ, underwent femoral artery ligation. Blood flow reperfusion was assessed up to 4 weeks post-surgery. Capillary density, EC apoptosis and VEGF signaling were evaluated in the ischemic muscle. Src homology region 2 domain-containing phosphatase-1 (SHP-1) phosphatase activity was assessed *in vitro* using primary ECs.

**Results:**

Ischemic muscle of diabetic *ec-Prkcd*^f/f^ mice exhibited reduced blood flow reperfusion and capillary density while apoptosis increased as compared to nondiabetic *ec-Prkcd*^f/f^ mice. In contrast, blood flow reperfusion and capillary density were significantly improved in diabetic *ec-Prkcd*^−/−^ mice. VEGF signaling pathway was restored in diabetic *ec-Prkcd*^−/−^ mice. The deletion of PKCδ in ECs prevented diabetes-induced VEGF unresponsiveness through a reduction of SHP-1 phosphatase activity.

**Conclusions:**

Our data provide new highlights in mechanisms by which PKCδ activation in EC contributed to poor collateral vessel formation, thus, offering novel therapeutic targets to improve angiogenesis in the diabetic limb.

## Key messages

- Endothelial deletion of PKCδ improve blood flow reperfusion in diabetic limb
- VEGF signaling is restored by the endothelial ablation of PKCδ
- PKCδ increase SHP-1 expression in endothelial cells of diabetic limb
- SHP-1 phosphatase activity is blunted in PKCδ-KO primary endothelial cells

## Introduction

Peripheral artery disease is a direct manifestation of obstructive atherosclerosis characterized by the progressive narrowing of the artery, which gradually reduces blood supply to the limbs causing critical ischemia.^1^ Diabetes increases the incidence of PAD and causes 10–16 times higher risk of non-traumatic lower limb amputation, mainly due to insufficient collateral vessel formation.^2^ Hyperglycemia has been shown to alter angiogenic processes involving complex interplays between various growth factors and cell types.^3^

Vascular endothelial growth factor (VEGF) plays a fundamental role in the angiogenic process. VEGF-induced stimulation of VEGF receptor 2 (VEGFR2) triggers the activation of downstream pathways that are involved in the migration and proliferation of endothelial cells (EC).^4^ Exogenous local injection of VEGF has been shown to improve neo-vascularization in both mice and humans in a nondiabetic state.^5,6^ However, local administration of VEGF by gene therapy in diabetic individuals did not appear to have the beneficial effects seen in the absence of diabetes.^7^ Decreased expression of VEGF was observed in the muscle of diabetic mice and correlated with impair revascularization.^8^ Similarly, we have shown that reduction of VEGF expression was associated with blunted phosphorylation of VEGFR2 and poor collateral vessel formation in the ischemic muscle of diabetic mice.^9^ These studies emphasized that impaired vessel formation following ischemia in a diabetic state can be partially attributed to both lack of production and unresponsiveness to critical growth factors including VEGF. Thus, the identification of the underlying molecular mechanisms involved in the deregulation of VEGF signaling in hyperglycemia has generated further interest.

Protein kinase C (PKC) has been identified as a major player in the pathophysiology of diabetic vascular complications.^10^ Hyperglycemia-induced activation of the PKCβ isoform contributed to the increase in EC adhesion molecule expression, which caused advanced development of atherosclerotic plaques in *ApoE*^−/−^ mice.^11^ Diabetes contributes to endothelial dysfunction by reducing the expression of endothelial nitric oxide synthase (eNOS) and thereby nitric oxide, an effect that can be prevented by the inhibition of PKC activity.^12^ Using a model of critical limb ischemia, we have reported that the PKCδ isoform was upregulated in muscle of diabetic mice.^13^ However, the precise contribution of PKCδ activation to EC dysfunction in diabetes and ischemia has never been fully investigated. Given the pivotal role of EC in angiogenesis, the present study investigated the role of PKCδ specifically in EC on blood flow reperfusion and vascular density in a context of hindlimb ischemia. We hypothesized that the endothelial deletion of PKCδ could restore VEGF signaling and promote collateral vessel formation to subsequently improve blood flow reperfusion in the ischemic diabetic limb.

## Materials and methods

### Reagents and antibodies

Primary antibodies for immunoblotting were purchased from commercial sources: actin (horseradish peroxidase (HRP); I-19), GAPDH-HRP (V18), SHP-1 (C19), VEGF (147), PDGFβ (N30) PLCγ (B10) and endothelial nitric oxide synthase (eNOS) 3 (C-20) from Santa Cruz Biotechnology Inc (Dallas, TX); protein kinase B (Akt), phospho-Akt (S473) (D9E), phospho-VEGFR2 (Y1175), VEGFR2, phospho-eNOS (S1177), phospho-SRC (Y416), SRC, phospho-PLCγ (Y783) and secondary antibody of anti-rabbit and -mouse peroxidase-conjugated from Cell Signaling (Danver, MA); anti-α smooth muscle actin from Abcam (Toronto, ON) and anti-CD31 and collagen type 1 for cell culture were purchased from BD Bioscience (Mississauga, ON). Secondary antibodies for immunofluorescence Alexa-488 conjugated anti-rabbit IgG and Alexa-594 conjugated anti-rat were purchased from Jackson ImmunoResearch Laboratories (West Grove, PA). Fetal bovine serum (FBS), phosphate-buffered saline (PBS), penicillin-streptomycin (P/S), Dynabead sheep anti-rat and Dulbecco’s Modified Eagle Medium (DMEM) low glucose (31600-034) were obtained from Invitrogen (Burlington, ON). VEGF-A_165_ was purchased from R&D (Minneapolis, MN). Collagenase type 1 was purchased from Wortington Biochemical Corporation (Lakewood, NJ). Endothelial cell growth supplement (ECGS) was purchased from Sciences Cells Research Laboratories. All other reagents were purchased from Sigma-Aldrich (St. Louis, MO).

### Animal and experimental design

Specific deletion of PKCδ in EC was generated by breeding *Prckd*^flox/flox^ mice^14^ with the VEC-CRE (B6;129-Tg(Cdh5-cre)1Spe/J) mice from Jackson laboratories (JAX stock #017968). Animals were rendered diabetic for a 2-month period by intraperitoneal streptozotocin injection (50 mg/kg in 0.05 mol/L citrate buffer, pH 4.5; Sigma) on five consecutive days after overnight fasting; control mice were injected with citrate buffer. Blood glucose was measured by Glucometer (Contour, Bayer Inc.). Throughout the study period, animals were provided with free access to water and standard rodent chow (Harlan Teklad, Madison, WI). All experiments were conducted in accordance with the Canadian Council of Animal Care and University of Sherbrooke guidelines.

### Hindlimb ischemia model

We have assessed blood flow in nondiabetic (NDM) and 4 month-old diabetic mice (DM), with (*ec-Prkcd*^−/−^) or without (*ec-Prkcd*^f/f^) specific deletion of PKCδ in ECs. Animals were anesthetized by inhalation of Isoflurane USP (1-chloro-2,2,2-trifluoroethyl difluoromethyl ether) at a concentration of 5% (initiation) and then maintained at 1%–2% during the whole surgical procedure (approximately 20 min) The entire lower extremity of each mouse was shaved. A small incision was made along the thigh all the way to inguinal ligament and extending superiorly towards the mouse abdomen. Femoral artery was isolated and ligated distally to the origin of the arteria profunda femoris as previously described.^13^

### Laser doppler perfusion imaging

Hindlimb blood flow was measured using a laser Doppler perfusion imaging (PIMIII) system (Perimed Inc) as previously described.^9^ Consecutive perfusion measurements were obtained by scanning the region of interest (hindlimb and foot) of anesthetized animals. Measurements were performed pre- and post-surgery to ensure the correct ligation, and additionally on postoperative days 7, 14, 21, and 28. To overcome the variables affecting blood flow temporally, results at any given time were expressed as a ratio of simultaneously obtained perfusion measurements of the right (ligated) versus the left (non-ligated) limb.

### Histology

Right and left adductor muscles from NDM; DM; *ec-Prkcd*^f/f^ and *ec-Prkcd*^−/−^mice were harvested for pathological examination and sections were fixed in 4% paraformaldehyde (Sigma) for 18 h and then transferred to 70% ethanol for light microscopy.

### Immunofluorescence and TUNEL assay

Cross-sections of adductor muscles of each group were blocked and then exposed overnight with primary antibodies (CD31 (1:50) and α-smooth muscle actin, 1:200), followed by 1h incubation with the secondary antibody (1:400). Apoptotic cells were detected using the TACS 2 Tdt-Fluor in situ apoptosis detection kit (Trevigen, Gaithersburg, MD), according to the manufacturer’s instructions with some modifications as we previously described.^9^ Images were taken with the Nikon eclipse Ti microscope. Three cross-sections, 50 µm apart, were taken per animal and all images were captured under identical settings and handled in Adobe Photoshop similarly across all images.

### Immunoblot analysis

Adductors muscles were lysed in RIPA buffer containing protease inhibitors.^9,13^ Protein quantity was measured with DC kit (BioRad). The lysates (20–50 µg protein) were separated by SDS-PAGE. Primary antibodies were incubated overnight at 1:1000 in 5% skim milk or 1:500 in 5% BSA for phospho-VEGFR2 and phospho-eNOS. Antigens were detected using anti-rabbit HRP-conjugated antibody 1:10 000 (or 1:2000 for phospho-VEGFR and phospho-eNOS) and detected with the either ECL system (Pierce Thermo Fisher, Piscataway, NJ) or Luminata forte western HRP substrate (Millipore, Etobicoke, ON). Protein content quantification was performed using computer-assisted densitometry with ImageLab imaging software (Chemidoc, BioRad).

### Quantitative PCR analysis

Quantitative PCR was performed to evaluate mRNA expressions of VEGF-A, KDR/Flk-1, eNOS, PDFG and PDGFR-β of the ischemic adductor muscle of all groups as we previously described.^13^ PCR primers are listed in Supplemental Table 1. GAPDH expression was used for normalization.

### Cell culture

Pulmonary EC were extracted from the lungs of each group of mice. Lungs were sliced into 1-2 mm pieces and incubated at 37°C for 1 h in 0.2% collagenase type 1. The tissue was disrupted by pipetting and passed through a 40 µm cell strainer. Following centrifugation, the pellet was resuspended in 1 ml DMEM/0.1% BSA and incubated with CD31-conjugated Dynabeads for 30 min at 4°C. Beads were wash with DMEM/0.1% BSA using a magnetic support and resuspended in growth medium (DMEM 10% FBS, 1% P/S, ECGS 50 µg/ml and heparin 1 mg/ml). Cells were plated in 100 mm petri dishes coated with type 1 collagen (0.1 mg/ml in 0.02 N acetic acid) and cultured in DMEM with normal (NG; 5.6 mmol/L + 19.4 mmol/L mannitol, NDM) or high (HG; 25 mmol/L, DM) glucose concentrations for 24 h. Cells were then stimulated or not with VEGF-A (10 ng/ml) for 10 min to assess Akt activation. Due to the limited amount of EC collected per mouse, bovine aortic EC (BAEC) were carefully isolated from aorta and alternatively used for phosphatase assays as previously described.^9^

### Adenoviral infections

Adenoviral vectors containing green fluorescent protein (GFP) and dominant negative form of PKCδ (Ad-dnPKCδ) or SHP-1 (Ad-dnSHP-1) were used to infect BAEC as we previously reported in retinal pericytes.^15^ BAEC were infected with adenoviral vectors at a multiplicity of infection of 100 for 48 h at 37°C.

### Phosphatase assay

SHP-1 was immunoprecipitated from cell lysates in buffer without phosphatase inhibitors as previously described.^9^ The tyrosine phosphatase assay system (V2471, Promega, Madison, WI) was used to assess phosphatase activity according to the manufacturer’s instructions.

### Statistical analyses

The in vivo and ex vivo data were shown as mean ± SD for each group. Statistical analysis was performed by one-way analysis of variance (ANOVA) followed by Tukey’s test correction for multiple comparisons. Data in each group were checked for normal distribution using D’Agostino and Pearson normality test based on α = 0.05. All results were considered statistically significant at *p* < 0.05.

## Results

### Specific deletion of PKCδ in EC improved blood flow reperfusion and vascular density in the diabetic muscle

**Figure 1.**
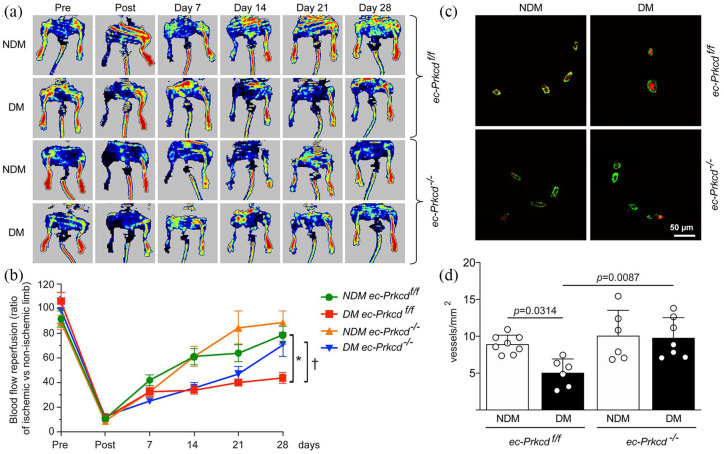
Blood flow reperfusion and vascular density in the ischemic limb of diabetic and nondiabetic mice: (a) laser Doppler imaging of nondiabetic (NDM) and diabetic (DM) *ec-Prkcd*^f/f^ and *ec-Prkcd*^−/−^ mice, pre, post, and 4 weeks following femoral artery ligation, (b) quantification of the percentage of blood flow reperfusion in the ischemic limb, (c) immunofluorescence of α-smooth muscle actin (green) and endothelial cells (CD31-red), and (d) quantification of vascular density in the ischemic muscle (number of vessel smaller than 30 μm) of three cross-sections per animal. Results are shown as mean ± SD of 12 (*ec-Prkcd*^−/−^) and 15 (*ec-Prkcd*^f/f^) mice per group (a and b) and 6–8 mice per group (c and d). *p = 0.0009 versus NDM *ec-Prkcd*^f/f^. †*p* = 0.0089 versus DM *ec-Prkcd*^−/−^.

Nondiabetic and diabetic *ec-Prkcd*^f/f^ and *ec-Prkcd*^−/−^ mice underwent unilateral femoral artery ligation. Body weight and fasting blood glucose levels were measured at euthanasia and the deletion of PKCδ did not affect both parameters in the context of diabetes (Supplemental Table 2). Blood flow reperfusion was assessed using the laser Doppler imaging system (Figure 1(a)). As expected, results showed a 47% decrease of blood flow reperfusion in diabetic *ec-Prkcd*^f/f^ (41% blood flow recovery) as compared to nondiabetic *ec-Prkcd*^f/f^ mice (78% blood flow recovery; *p* = 0.0009). The specific deletion of PKCδ in EC significantly improved blood flow reperfusion in the ischemic limb of diabetic *ec-Prkcd*^−/−^ mice as compared to diabetic *ec-Prkcd*^f/f^ mice (*p* = 0.0089) (Figure 1(b)). Importantly, diabetic *ec-Prkcd*^f/f^ mice displayed a significant 37.5% reduction of vascular density (*p* = 0.0314) as compared to nondiabetic *ec-Prkcd*^f/f^ mice, suggesting impaired collateral vessel formation (Figure 1(c) and (d)). In contrast, enhanced blood flow reperfusion observed in diabetic *ec-Prkcd*^−/−^ mice was associated with a 50% increase in vascular density (*p* = 0.0087) as compared to diabetic *ec-Prkcd*^f/f^ mice.

### PKCδ deletion preserved muscle integrity and prevented EC apoptosis in the ischemic diabetic limb

**Figure 2.**
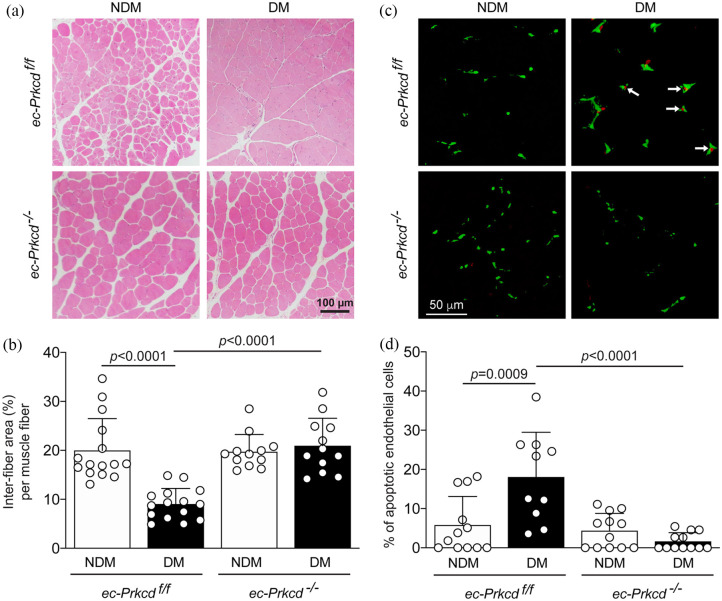
Histological analysis of muscle integrity and endothelial cells apoptosis: (a) histological cross-sections of diabetic and nondiabetic ischemic muscle stained with hematoxylin-eosin, (b) quantification of the percentage of the inter-fiber area per muscle fiber, (c) immunofluorescence of α-smooth muscle actin (green) and TUNEL endothelial positive cells (CD31-red) in the ischemic muscle of nondiabetic and diabetic *ec-Prkcd*^f/f^ and *ec-Prkcd*^−/−^ mice, and (d) quantification of the percentage of endothelial apoptotic cells per mm² in the cross-sections of ischemic muscle in each group. Results are shown as mean ± SD of 12–15 mice per group (a and b) and 10–12 mice per group (c and d).

Muscle integrity was significantly (*p* < 0.0001) altered in diabetic *ec-Prkcd*^f/f^ mice as compared to nondiabetic *ec-Prkcd*^f/f^ mice, which was characterized by the reduction of the inter-fiber area (Figure 2(a) and (b)). Histological observations showed complete preservation of the muscle integrity and structure 28 days post-ligation in diabetic *ec-Prkcd*^−/−^ mice, which displayed similar muscle fiber organization as nondiabetic *ec-Prkcd*^f/f^ mice (Figure 2(a) and (b)). Another potential mechanism that could explain poor blood flow reperfusion in diabetic mice might be due to early vascular cell apoptosis during ischemia. Therefore, we have quantified the number of positive apoptotic-CD31 co-stained cells in the ischemic muscle of each group (Figure 2(c) and (d)). The percentage of positive apoptotic EC was significantly increased (*p* = 0.0009) in diabetic *ec-Prkcd*^f/f^ mice as compared to nondiabetic *ec-Prkcd*^f/f^ mice. Interestingly, the deletion of PKCδ in diabetic mice greatly reduced the percentage of apoptotic cells to similar levels as nondiabetic *ec-Prkcd*^f/f^ mice.

### Inhibition of PKCδ specifically in EC restored VEGFR2 but not PDGFR-β expression

**Figure 3.**
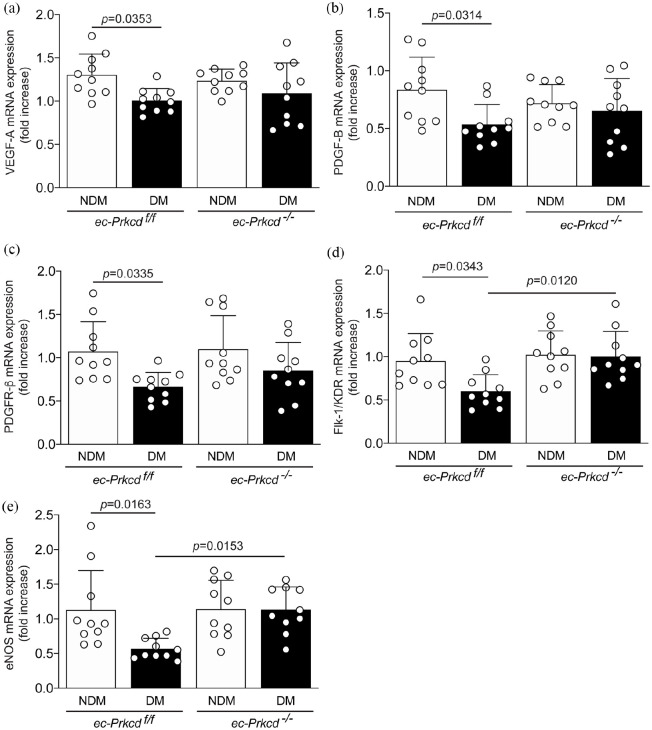
Decreased mRNA expression of angiogenic factors and eNOS in the muscle of diabetic *ec-Prkcd*^f/f^ mice. Quantitative real-time PCR of: (a) VEGF-A, (b) PDGF-B, (c) PDGF-β, (d) FLK-1, and (e) eNOS in ischemic muscle of nondiabetic (NDM) and diabetic (DM) *ec-Prkcd*^f/f^ and *ec-Prkcd*^−/−^ mice. Results are shown as mean ± SD of 10–12 mice per group.

We have demonstrated that whole body PKCδ knockout mice prevented diabetes-induced inhibition of VEGF and PDGF expression.^13^ Since several cell types in the muscle can participate in the angiogenic response, we have investigated the endothelial PKCδ contribution to the expression of pro-angiogenic factors. Both VEGF-A and PDGF-B mRNA expression were significantly decreased in the muscle of diabetic mice by respectively 30 and 50% as compared to nondiabetic *ec-Prkcd*^f/f^ animals (Figure 3(a) and (b)). However, mRNA expressions were not fully restored in diabetic *ec-Prkcd*^−/−^ mice as compared to diabetic *ec-Prkcd*^f/f^ mice suggesting that PKCδ in EC is not responsible for the expression of VEGF-A and PDGF-B in the muscle. Similarly, PDGFR-β mRNA expression was significantly blunted in diabetic *ec-Prkcd*^f/f^ mice and remained decreased with the absence of PKCδ in EC (Figure 3(c)). Interestingly, VEGFR2 mRNA was restored by 60% (*p* = 0.0314) in diabetic *ec-Prkcd*^−/−^ mice as compared to diabetic *ec-Prkcd*^f/f^ mice (Figure 3(d)). The expression of eNOS is essential for EC function and its activity is regulated in part by VEGFR2. Our data demonstrated that diabetes reduced the mRNA expression of eNOS in the ischemic diabetic muscle, which was restored with the ablation of PKCδ specifically in EC (Figure 3(e)).

### VEGFR2 activation and signaling pathways are restored in diabetic endothelial-specific PKCδ null mice

**Figure 4.**
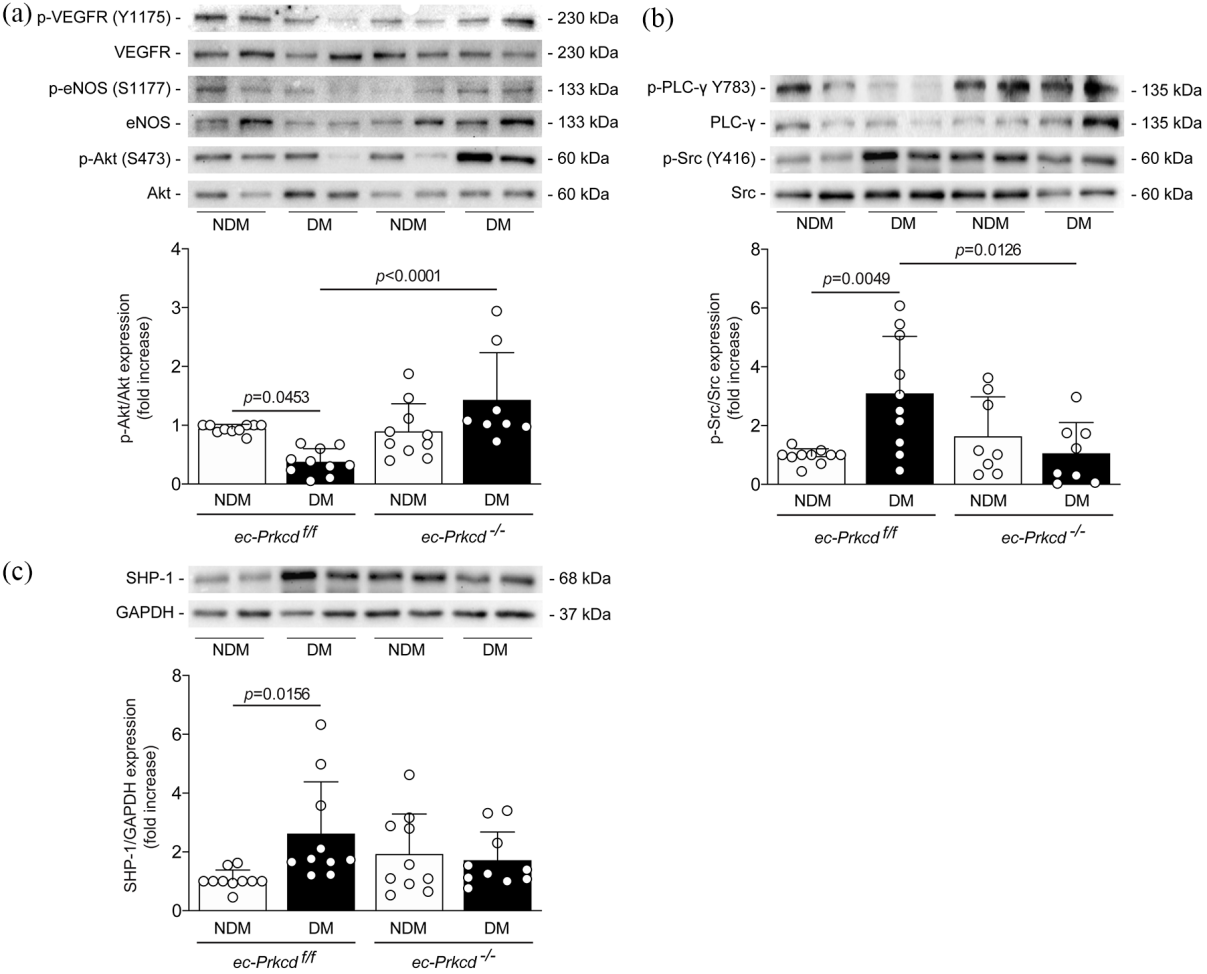
Decreased activation of the VEGF signaling pathway and increased expression of SHP-1 in the muscle of diabetic *ec-Prkcd*^f/f^ mice: (a) expression of phospho-VEGFR (Y1175), VEGFR, phospho-eNOS (S1177), eNOS, phospho-Akt (S473), (b) phospho-PLC-γ (Y783), PLC-γ, phospho-Src (Y416), Src, and (c) SHP-1 and GAPDH in ischemic muscle of nondiabetic (NDM) and diabetic (DM) *ec-Prkcd*^f/f^ and *ec-Prkcd*^−/−^ mice. Protein expression was detected by immunoblot blot, and densitometric quantitation was measured. Results are shown as mean ± SD of 8–10 mice per group.

Since VEGFR2 expression was restored in diabetic *ec-Prkcd*^−/−^ mice, we have investigated the downstream signaling pathways of VEGF in the ischemic muscle. Our results indicated that the activation of VEGFR2 was reduced in diabetic *ec-Prkcd*^f/f^ animals and fully restored in diabetic *ec-Prkcd*^−/−^ mice (Figure 4(a)). One of the downstream targets of VEGFR2 is the phosphorylation of Akt, which promotes cell survival. The activation of Akt was significantly reduced by 50% (*p* = 0.0453) in the diabetic ischemic muscle of *ec-Prkcd*^f/f^ mice and completely prevented with the ablation of PKCδ specifically in EC (Figure 4(a)). Activation of Akt leads to eNOS phosphorylation in EC. Similar to eNOS mRNA expression, eNOS phosphorylation was abrogated in the ischemic muscle of diabetic *ec-Prkcd*^f/f^ mice and the endothelial-specific deletion of PKCδ maintained eNOS phosphorylation in the ischemic muscle (Figure 4(a)). PLCγ, a downstream regulator of the VEGF pathway, exhibited a similar pattern as eNOS expression (Figure 4(b)). In contrast, Src activation was significantly (*p* = 0.0049) enhanced in the ischemic muscle of diabetic *ec-Prkcd*^f/f^ mice as compared to nondiabetic *ec-Prkcd*^f/f^ mice. The ablation of PKCδ in EC led to a significant (*p* = 0.0126) reduction of Src phosphorylation in the ischemic muscle of diabetic *ec-Prkcd*^−/−^ mice (Figure 4(b)).

### SHP-1 expression and phosphatase activity were increased in the muscle of diabetic mice and abrogated with the inhibition of PKCδ

**Figure 5.**
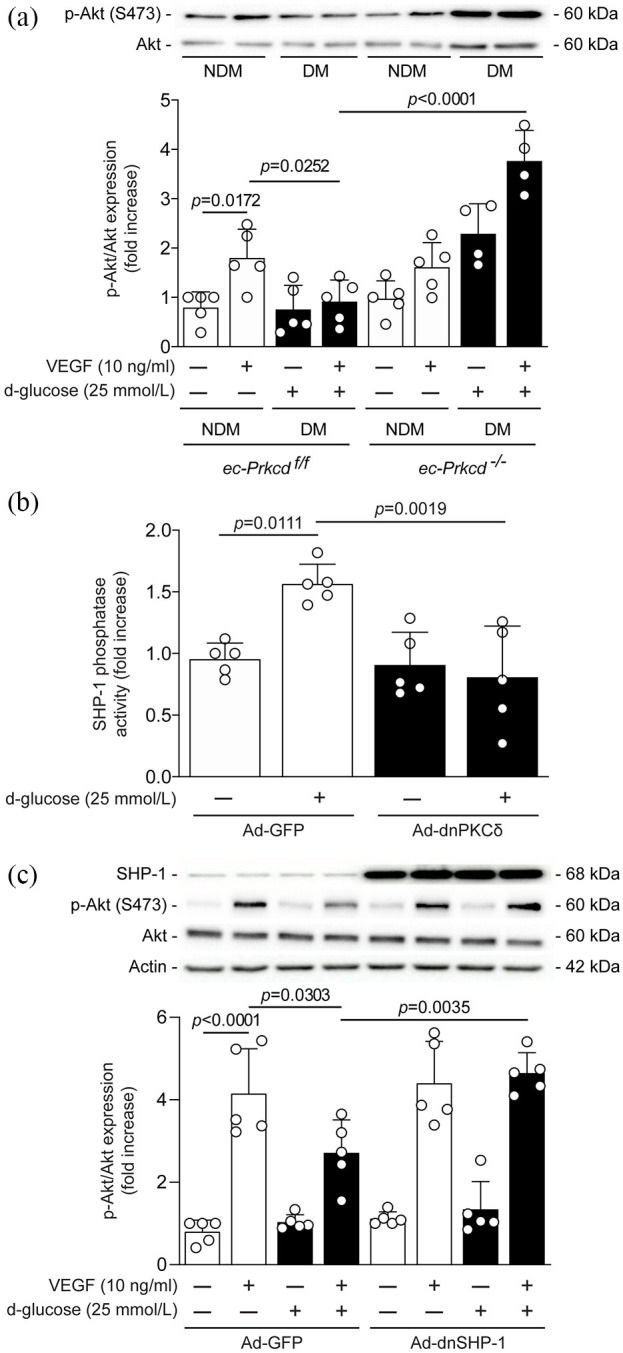
Akt expression and activation in lung EC and SHP-1 phosphatase activity in BAEC: (a) expression of phospho-Akt (S473) and total Akt in EC isolated from the lungs of diabetic (DM) and nondiabetic (NDM) *ec-Prkcd*^f/f^ and *ec-Prkcd*^−/−^ mice exposed to NG (clear bars) or HG (black bars) concentrations followed by VEGF stimulation. Protein expression was detected by immunoblot blot, and densitometric quantitation was measured, (b) phosphatase activity of SHP-1 in BAEC infected or not with the adenoviral vector of dominant negative form of PKCδ, and (c) expression of phospho-Akt (S473), total Akt in BAECs infected with either Ad-GFP or Ad-dnSHP-1 and exposed to NG (clear bars) or HG (black bars) concentrations followed by VEGF stimulation. Protein expression was detected by immunoblot blot, and densitometric quantitation was measured. Results are shown as mean ± SD test of 4 (a) and 5 (b, c) independent experiments.

We have previously demonstrated that PKCδ activation was associated with increased SHP-1 expression in retinal pericytes^15^ and renal epithelial cells of diabetic mice.^16^ Our data demonstrated a significant 2.5-fold increase (*p* = 0.0156) of SHP-1 expression in the ischemic muscle of diabetic *ec-Prkcd*^f/f^ mice (Figure 4(c)). SHP-1 expression was reduced by 58% in the ischemic muscle of diabetic *ec-Prkcd*^−/−^ mice as compared to diabetic *ec-Prkcd*^f/f^ mice but it was not statistically significant (*p* = 0.2122). Due to cell heterogenicity that compose the skeletal muscle, EC were extracted from the lungs of each group and then stimulated with VEGF-A. First, we confirmed ex vivo that the activation of Akt was twice as high in VEGF stimulated EC from nondiabetic *ec-Prkcd*^f/f^ mice as compared to non-stimulated cells (*p* = 0.0172) and blunted in EC from diabetic *ec-Prkcd*^f/f^ mice (Figure 5(a)). In contrast, the phosphorylation of Akt was enhanced in EC from diabetic *ec-Prkcd*^−/−^ mice (*p* < 0.0001) as compared to diabetic *ec-Prkcd*^f/f^ mice following VEGF stimulation (Figure 5(a)). Since it has been shown that enhanced SHP-1 phosphatase activity, rather than expression, decreased VEGF signaling,^9^ we investigated if this increase could be dependent on PKCδ activation. Due to technical limitations involving an insufficient amount of lung ECs from mice, BAEC were infected with adenoviral vector containing the dominant negative form of PKCδ in phosphatase assay. Interestingly, SHP-1 phosphatase activity was significantly increased by 50% (*p* = 0.0111) in BAEC exposed to HG levels as compared to NG, whereas the overexpression of the dominant negative form of PKCδ significantly prevented SHP-1 phosphatase activity in HG conditions (*p* = 0.0019) (Figure 5(b)). The role of SHP-1 in PKCδ-induced VEGF inhibition was also explored. As expected, VEGF-stimulated BAECs exhibited a 4-fold increase in the activation of Akt as compared to non-stimulated cells (*p* < 0.0001), an effect that was significantly decreased by 35% by HG level exposure (*p* = 0.0303). Interestingly, the overexpression of the dominant negative form of SHP-1 in BAECs exposed to HG levels completely restored VEGF-induced phosphorylation of Akt (*p* = 0.0035), an effect that was blunted in control GFP-overexpressed BAECs (Figure 5(c)).

## Discussion

PAD is more frequent, aggressive and occurs younger in diabetic individuals, which causes worse outcomes and increases the risk of lower limb amputations. Although hyperglycemia is known to alter VEGF-induced angiogenic responses, the underlying molecular mechanisms of reduced VEGF expression and activity in diabetic PAD remain unclear. We have previously uncovered the involvement of the PKCδ isoform in the inhibition of VEGF signaling in the whole ischemic muscle of diabetic mice.^13^ In our current study, we demonstrated for the first time that hyperglycemia-induced activation of PKCδ specifically in EC reduced VEGF actions. The ablation of PKCδ only in EC was able to restore VEGFR2 activity and blood flow reperfusion following ischemia of a diabetic limb.

Angiogenesis is a complex, dynamic and well-coordinated physiological process leading to the formation of new vessels from pre-existing vasculature in response to ischemia.^17^ EC play a crucial role in the initialization of the angiogenic response to the development of mature and functional capillaries. Several studies have shown that hyperglycemia contributes to the activation of several PKC isoforms, especially PKCβ, drastically altering EC metabolism^18^ and subsequently blood flow reperfusion following ischemia.^19^ Our study demonstrated for the first time that PKCδ is responsible for EC dysfunction during ischemia and diabetes. In addition, blood flow reperfusion was completely restored in diabetic *ec-Prkcd*^−/−^ mice, indicating that the specific activation of PKCδ in EC was sufficient to alter blood reperfusion in the ischemic diabetic limb. Improved blood flow reperfusion observed in diabetic *ec-Prkcd*^−/−^ mice was associated with increased density of small vessels (diameter < 10 µm) and arterioles (<30 µm). Hyperglycemia has been shown to affect EC proliferation,^20^ migration^9^ and tubule formation,^21^ three significant steps of the angiogenic process. Therefore, deletion of PKCδ in EC may promote angiogenesis by restoring EC function in a diabetic state. A shared factor of many diabetic vascular complications is the enhanced rate of cellular apoptosis^15^ Therefore, impaired blood flow reperfusion could be due to early EC apoptosis during ischemia. Diabetes increased EC apoptosis in the ischemic muscle, which was reduced by the deletion of PKCδ specifically in EC.

Both VEGF-A and PDGF-B play a complementary role in the regulation of the angiogenic response to ischemia. VEGF is involved in the early steps of angiogenesis^22^ while PDGF is required for the maturation and stabilization of newly formed vessels.^23^ We observed a decrease in the gene expression of both VEGF-A and PDGF-B in the ischemic muscle of diabetic mice. These results were consistent with previous findings using rat and rabbit models.^24^ However, the expression of VEGF-A, PDFG-B, and PDGFR-β was not restored by the endothelial-specific deletion of PKCδ. Given that perivascular cells are also known to produce VEGF and PDGF,^25^ the endothelial-specific deletion of PKCδ could have been insufficient to compensate for decreased expression of pro-angiogenic factors in the whole adductor muscle. Likewise, as PDGFR-β is mainly expressed in vascular smooth muscle cells,^26^ the absence of PKCδ only in EC may not be sufficient to influence the PDGFR-β expression in the entire muscle tissue. However, this hypothesis will require further investigation.

The alteration of VEGFR2 activation is associated with an impaired angiogenic response. Previous studies have shown that high glucose levels reduced VEGFR2 expression and activation in primary EC^27^ and diabetic mice.^9^ Our data demonstrated that the endothelial-specific deletion of PKCδ preserved VEGFR2 phosphorylation. Akt and eNOS are key downstream effectors of the VEGF pathway that promote EC survival,^28^ permeability, and migration.^29^ Interestingly, activation of Akt and eNOS was recovered in the ischemic muscle of diabetic mice lacking PKCδ only in EC. Another study reported that the inhibition of PKCδ promoted metabolic activity and expansion of the hematopoietic stem and progenitor cells partially through the PI3K/Akt pathway.^30^ PKCδ-induced alteration of EC function in diabetes was corroborated by a similar Akt inhibition pattern observed in isolated EC from the lungs of diabetic *ec-Prkcd*^f/f^ mice. The reestablishment of Akt activation in EC of diabetic *ec-Prkcd*^−/−^ mice confirmed the involvement of PKCδ in diabetes-induced inhibition of the VEGF signaling pathway.

Protein tyrosine phosphatases are critical to downregulate cell response to growth factors by decreasing receptor tyrosine kinase phosphorylation.^31^ It has been reported that SHP-1 negatively regulated the VEGF pathway by inhibiting EC proliferation in vitro.^32^ The inhibition of SHP-1 increased VEGFR2 phosphorylation and enhanced capillary density in a nondiabetic ischemic rat model.^33^ Hyperglycemia-induced SHP-1 overexpression has been shown to interact with nephrin in podocytes^34^ and PDGFR^15^ in retinal pericytes leading to the progression of diabetic nephropathy and retinopathy, respectively. In the context of PAD, our data showed that SHP-1 expression was increased in the muscle of diabetic animals. The deletion of PKCδ specifically in EC did not significantly reduce SHP-1 expression suggesting that other cells might have contributed to the upregulation of SHP-1 in the entire muscle. On the other hand, increased phosphatase activity of SHP-1 in EC exposed to high glucose levels was abrogated by the overexpression of a dominant negative form of PKCδ. Our data suggest that PKCδ regulates SHP-1 activity rather than its expression. Furthermore, inhibition of SHP-1 restored VEGF-induced Akt activation in EC exposed to high glucose concentrations. While these results do not establish a direct connection between SHP-1 and PKCδ, it at least confirmed that SHP-1 is involved in impaired VEGF signaling actions caused by hyperglycemic condition in EC. Nevertheless, additional experiments will be required to determine if the effects of SHP-1 deletion are dependent of the activation of PKCδ in a diabetic EC. Another limitation of our study is that PKCδ is known to affect other important angiogenic pathways such as PDGF^15,35^ and angiotensin II^36,37^ signaling. Therefore, the improvement of angiogenesis observed by the ablation of PKCδ in EC could also have been partially due to the reestablishment of the above-mentioned pro-angiogenic pathways.

In summary, our study provided new insights on diabetes-induced PKCδ activation specifically in EC, which was shown to contribute to the inhibition of VEGFR2 signaling through the increase in SHP-1 phosphatase activity. Our data enhance our knowledge of the molecular mechanisms underlying poor collateral vessel formation induced by diabetes and highlight PKCδ as a potential pharmacological target to treat PAD in diabetic patients. However, potential therapeutic modulation of PKCδ in patients with diabetes and PAD needs to be locally selective to EC in ischemic muscle as pathological events may arise from systemic treatment. Indeed, PKCδ isoform is involved in several cardiovascular diseases^10^ and systemic deletion of PKCδ has been shown to accelerate arteriosclerotic lesions in mice^35^ and enhance the expression of connective tissue growth factor in cardiomyocytes leading to the progression of cardiac fibrosis in diabetic cardiomyopathy.^36^

## Supplemental Material

sj-pdf-1-dvr-10.1177_1479164121999033 – Supplemental material for Endothelial deletion of PKCδ prevents VEGF inhibition and restores blood flow reperfusion in diabetic ischemic limb Click here for additional data file. Supplemental material, sj-pdf-1-dvr-10.1177_1479164121999033 for Endothelial deletion of PKCδ prevents VEGF inhibition and restores blood flow reperfusion in diabetic ischemic limb by Laura Croteau, Clément Mercier, Étienne Fafard-Couture, Alexandre Nadeau, Stéphanie Robillard, Valérie Breton, Andréanne Guay, Farah Lizotte, Marc-Antoine Despatis and Pedro Geraldes in Diabetes & Vascular Disease Research
