# Supplementary material for: Endothelial deletion of PKCδ prevents VEGF inhibition and restores blood flow reperfusion in diabetic ischemic limb
Source: Diab Vasc Dis Res. 2021 Mar 15;18(2):1479164121999033. doi: 10.1177/1479164121999033 (PMC8481738; doi:10.1177/1479164121999033)
Supplement: sj-pdf-1-dvr-10.1177_1479164121999033 – Supplemental material for Endothelial deletion of PKCδ prevents VEGF inhibition and restores blood flow reperfusion in diabetic ischemic limb [file sj-pdf-1-dvr-10.1177_1479164121999033.pdf]

# Supplementary Material

# Endothelial deletion of PKCδ prevents SHP-1-induced VEGF inhibition and restores angiogenesis in diabetic ischaemic limb

Laura Croteau[1*], Clément Mercier[1*], Étienne Fafard-Couture[1], Alexandre Nadeau[1], Stéphanie Robillard[1], Valérie Breton[1], Andréanne Guay[1], Farah Lizotte[1], Marc-Antoine Despatis[2] and Pedro Geraldes.[1,3△]

**Table 1**. Sequences of primers

| Gene | Forward | Reverse |
|---|---|---|
| mVEGF-A | GGAGTACCCCGACGAGATAGAGTA | AGCCTGCACAGCGCATC |
| mKDR/Flk-1 | AGAACACCAAAAGAGAGAGGAACG | GCACACAGGCAGAAACCAGTAG |
| meNOS | GTTTGTCTGCGGCGATGTC | GAATTCTCTGCACGGTTTGCA |
| mPDGF-B | TGAAATGCTGAGCGACCACTCCAT | AGTGTGCTCGGGTCATGTTCAAGT |
| mPDGFR-β | ACAAGACCTACATCTGCAAAACCA | TCACCCTGGAGGCTGTAGACAT |

**Table 2**. Body weight and blood glucose levels

| | *ec-Prkcd* $^{f/f}$ | | *ec-Prkcd* $^{-/-}$ | |
|---|---|---|---|---|
| | NDM | DM | NDM | DM |
| Body weight (g) | 27.2 ± 2.8 | 21.0 ± 3.5 | 24.8 ± 2.0 | 21.9 ± 2.0 |
| Blood glucose levels (mg/dl) | 169 ± 50 | 520 ± 124 | 158 ± 32 | 543 ± 94 |
